# Pathophysiological role of ion channels and transporters in gastrointestinal mucosal diseases

**DOI:** 10.1007/s00018-021-04011-5

**Published:** 2021-11-15

**Authors:** Zilin Deng, Yingying Zhao, Zhiyuan Ma, Minglin Zhang, Hu Wang, Zhiqiang Yi, Biguang Tuo, Taolang Li, Xuemei Liu

**Affiliations:** 1 Department of Gastroenterology, Affiliated Hospital of Zunyi Medical University, Zunyi, 563003 Guizhou Province China; 2 Department of Thyroid and Breast Surgery, Affiliated Hospital of Zunyi Medical University, Zunyi, 563003 Guizhou Province China

**Keywords:** Ion channels and transporters, Mucosal barrier, Mucosal diseases, Repair

## Abstract

The incidence of gastrointestinal (GI) mucosal diseases, including various types of gastritis, ulcers, inflammatory bowel disease and GI cancer, is increasing. Therefore, it is necessary to identify new therapeutic targets. Ion channels/transporters are located on cell membranes, and tight junctions (TJs) affect acid–base balance, the mucus layer, permeability, the microbiota and mucosal blood flow, which are essential for maintaining GI mucosal integrity. As ion channel/transporter dysfunction results in various GI mucosal diseases, this review focuses on understanding the contribution of ion channels/transporters to protecting the GI mucosal barrier and the relationship between GI mucosal disease and ion channels/transporters, including Cl^−^/HCO_3_^−^ exchangers, Cl^−^ channels, aquaporins, Na^+^/H^+^ exchangers, and K^+^ channels. Here, we provide novel prospects for the treatment of GI mucosal diseases.

## Introduction

The gastrointestinal (GI) mucosa is a defense barrier against many harmful and immunogenic substances in the GI tract. The gastric mucosa lining the mucus-bicarbonate border comprises a continuous layer of epithelial cells connected by tight junctions (TJs) and blood vessels that supply oxygen and nutrients [1]. A key aspect of the acid resistance of the gastric mucosa involves the diffusion of bicarbonate produced by parietal cells to the mucous layer [2]. The first layer of the intestinal barrier consists of the flora in the cavity. The second layer is a microenvironment composed of an unstirred water layer, the glycocalyx, and a mucus layer. The third layer comprises intestinal epithelial connected by TJs and immune cell secretions in the lamina propria [3]. Acid–base imbalance, bacterial infection, mucus layer damage, and microbial dysbiosis lead to mucosal diseases, such as peptic ulcer, hypergastrinemia, autoimmune gastritis, GI tumors and inflammatory bowel disease (IBD) [4–7].

**Fig. 1:**
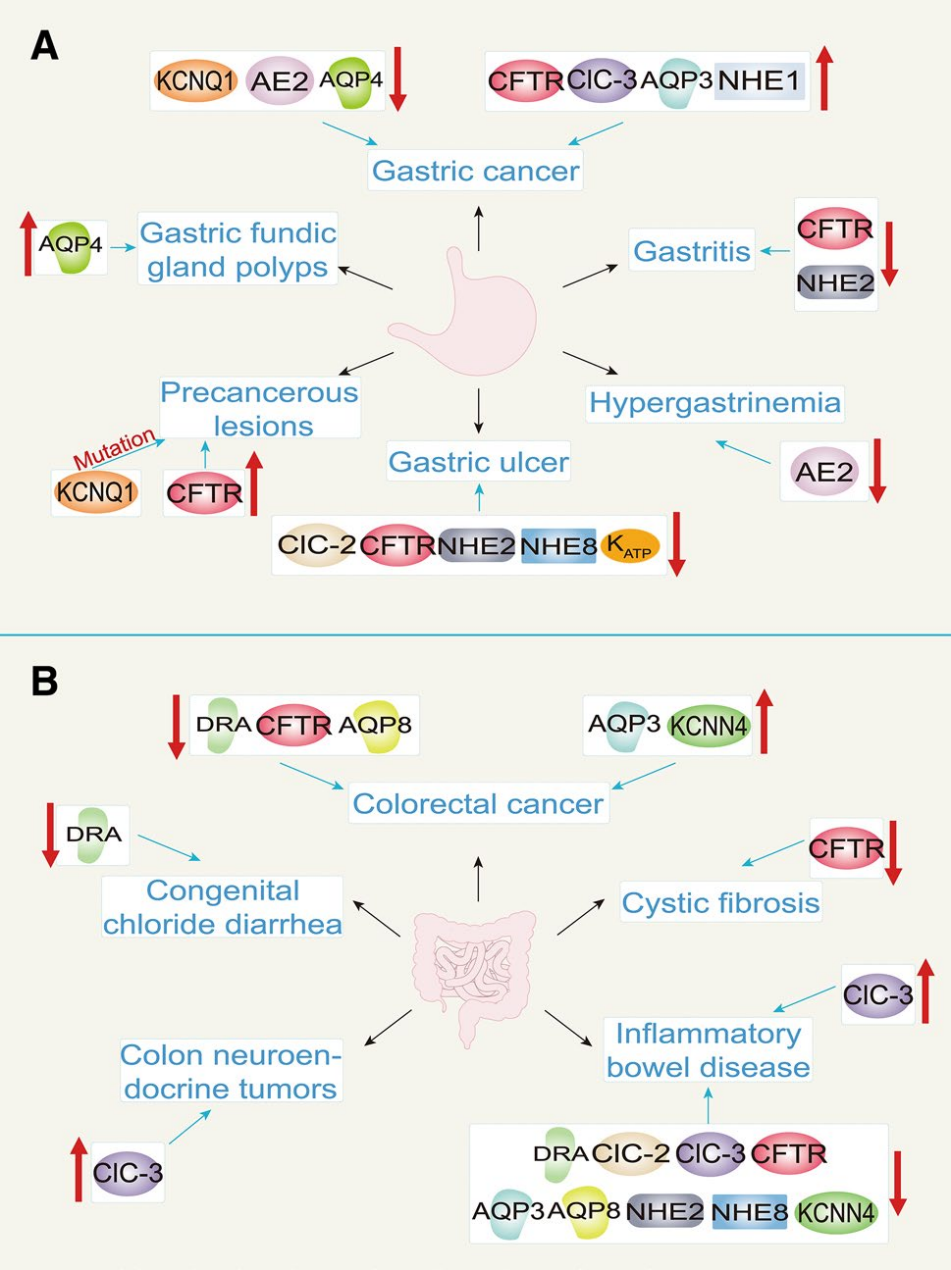
Dysfunction of ion channels and transporters resulted in gastrointestinal mucosal diseases. **A** Gastric mucosal diseases. **B** Intestinal mucosal diseases. (The upward arrows represent upregulation or activation of ion channels/transporters, and the downward arrows represent downregulation or inhibition of ion channels/transporters)

Ion channels and transporters embedded in the cell membrane are essential for maintaining acid–base balance [8]. The stomach needs to withstand the high gastric acid environment caused by parietal cells, a condition that increases the probability of gastric mucosal damage and can even cause perforation [9]. The key to gastric mucosal resistance to acid is the production of bicarbonate; indeed, regardless of how much acid is produced, the corresponding amount of bicarbonate can neutralize it [2]. Loss of ion channels and transporters causes GI mucosal injury, such as bicarbonate and mucous layer destruction [10–13], epithelial cell loss [14, 15], glandular mucosal atrophy [16], TJ protein loss [17–19], flora imbalance [20] and mucosal blood flow changes [21]. Thus, ion channels and transporters play important roles that directly affect the mucosa, as well as TJs, microbial distribution, and mucosal blood flow. In addition, ion channels and transporters are the most important component of acid–base equilibrium and closely related to mucosal diseases. This review summarizes recent studies focusing on the pathophysiological role of ion channels and transporters in GI mucosal diseases, including Cl^−^/HCO_3_^−^ exchangers, Cl^−^ channels, aquaporins, Na^+^/H^+^ exchangers, and K^+^ channels. (Table 1, Fig. 1).

**Table 1:** The pathophysiological role of ion channels and transporters in gastrointestinal mucosal diseases is mentioned in this review

Ion channels/transporters	Genes	Other name	Digestive organ	Cell localization	Physiological function	Pathological conditions caused by dysfunction
Cl^−^/HCO_3_^−^ exchangers	SLC4A2	AE2	Stomach	Basolateral	Responsible for normal acid secretion	The occurrence of GC and the lack of acid secretion are related to the downregulation of AE2
	SLC26A3	DRA	Colon	Apical	Maintain a stable bicarbonate barrier and TJs between cells	Mutation of DRA gene can cause CLD, while downregulation of DRA can cause IBD and CRC
Cl^−^ channels	CLCN2	ClC-2	Stomach	Apical	Uncertain whether it is involved in gastric acid secretion, but it can protect intestinal TJs	ClC-2 promotes GI inflammation and tumorigenicity
			Colon	Basolateral		
	CLCN3	ClC-3	Stomach	Apical	Involved in regulating cell volume, the cell cycle, apoptosis, and cell migration	ClC-3 participates in inflammation and induces GI tumors
			Colon			
	ABCC7	CFTR	Stomach	Apical	CFTR secretes bicarbonate and participates in maintaining normal GI mucus secretion	CFTR can directly cause CF and GI cancers
			Colon			
Aquaporins	AQP3	AQP3	Colon	Basolateral	Transfer water and glycerin and maintain the integrity of TJ	AQP3 is involved in the process of IBD and GI cancer
	AQP4	AQP4	Stomach	Basolateral	It is uncertain whether AQP4 involved in gastric acid secretion, but closely related to the degree of parietal cell regeneration	AQP4 is downregulated in inflammation and GC, but upregulation of AQP4 can cause sporadic FGP
	AQP8	AQP8	Colon	Apical	AQP8 can maintain normal water flux and mucus viscosity	Downregulation of AQP8 is closely related to IBD and CRC
Na^+^/HCO_3_^−^ exchangers	SLC9A1	NHE1	Stomach	Basolateral	Facilitate cell migration	NHE1 is upregulation in GC and promote 5-Fu resistance
	SLC9A2	NHE2	Stomach	Basolateral	NHE2 is one of the targets of TFF to promote gastric epithelial repair and maintain the integrity of the intestinal TJ	Downregulation of NHE2 can lead to peptic ulcer, ulcer recurrence and IBD
			Colon	Apical		
	SLC9A8	NHE8	Stomach	Apical	Maintain normal bicarbonate secretion in the GI tract	Downregulation of NHE8 can cause intestinal inflammation and tumorigenicity
			Colon			
K^+^ channels	KCNJ8	K_ATP_ K_ir_6.1	Stomach	Vascular endothelial cells	Maintain normal gastric mucosal blood flow	K_ATP_ may be involved in gastric damage and the occurrence of gastric ulcer
	KCNQ1	K_V_7.1	Stomach	Apical	KCNQ1 participates in K^+^ recycling and gastric acid secretion	KCNQ1 is involved in the occurrence and development of precancerous lesions and GC
	KCNN4	K_Ca_3.1	Intestine	Apical	Participate in assisting HCO_3_^−^ and Cl^−^secretion and regulate T cell activation	KCNN4 is involved in the occurrence and development of IBD and CRC, and may be a target for CRC drug resistance treatment
				Basolateral

## Cl^−^/HCO_3_^−^ exchangers

Both the anion exchanger (AE) family (also known as the SLC4 family) and solute carrier 26 (SLC26) family mediate the transport of Cl^−^ and HCO_3_^−^. Members the AE family are Cl^−^/HCO_3_^−^ exchanger proteins independent of Na^+^ transporters and have vital roles in regulating cell volume and maintaining intracellular pH [22]. Members of the SLC26 family are the second-largest membrane proteins encoded by the human genome, including ten genes (SLC26A1–A11; SLC26A10 is a pseudogene) that are responsible for various monovalent and divalent anion transmembrane transport pathways, affecting the composition and pH of secreted fluids in the body. Among them, SLC26A1, -2, -3, -6, -7, -9, and -11 are expressed on the apical or basolateral membrane of GI epithelial cells [23–25].

### Expression pattern and functional role of Cl^−^/HCO_3_^−^ exchangers in the GI tract

#### AE2 in the GI tract

The AE family (SLC4A1–3) includes three subtypes: AE1, AE2, and AE3. AE2 is highly expressed on the basolateral membrane of gastric parietal cells, especially parietal cells in the neck of gastric glands [26]. Its activity is closely related to parietal cell secretion. AE2 mediates exchange of Cl^−^ and HCO_3_^−^, not only neutralizing luminal H^+^ but also providing Cl^−^ for apical membrane secretion [27, 28]. AE2 activity is considered the main mechanism for the outflow of HCO_3_^−^ and the inflow of Cl^−^ in acid secretion on the basolateral membrane. Mice lacking AE2 show reduced gastric acid and parietal cell numbers. Pathological conditions such as moderate expansion of the gastric gland cavity, severely impaired secretory tubule development, impaired secretory canaliculi, and decreased tubulovesicles also occur. Therefore, AE2 is necessary for parietal cells to secrete gastric acid [29]. Although AE2 (a, b), one of the AE2 mRNA variants, does not affect basal acid secretion when it losses, but significantly reduces acid secretion after carbachol/histamine stimulation [27]. NH_4_^+^ can regulate AE. In an acidic environment, activation of AE2 by NH_4_^+^ helps to maintain Cl^−^/HCO_3_^−^ exchange activity [30]. Carbonic anhydrase IX (CAIX) colocalizes with AE2 at the basolateral membrane, forming a bicarbonate transport complex. Interaction between the extracellular catalytic domain of CAIX and AE2 maximizes the acid secretion capacity of parietal cells. CAIX reacts CO_2_ with H_2_O to produce H^+^ and HCO_3_^−^, providing the required H^+^ for apical membrane secretion; AE2 provides Cl^−^ and pumps out excess HCO_3_^−^ to maintain the acid–base balance in parietal cells. The catalytic domain of CAIX binds to AE and enhances transmembrane HCO_3_^−^ flux [31]. In addition, CAIX and AE2 interaction promotes cell migration by controlling the pH of the protruding fronts of moving cells [32].

#### SLC26A3 in the intestinal tract

SLC26A3 (DRA), which is highly expressed on the apical membrane of the ileum and colon, is closely related to bicarbonate secretion, a stable mucus layer, and the mucosal barrier. Mice lacking DRA exhibit a weakened mucus layer and low HCO_3_^−^ secretion rate [10, 33]. DRA also directly binds to the TJ protein of intestinal epithelial cells, which can stabilize the structure of TJ and reverse the effect of tumor necrosis factor-α (TNF-α) on mechanical barrier damage. Even in the presence of TNF-α in IBD, cells overexpressing DRA show significantly increased levels of ZO-1 and occludin [17]. A recent report indicated that DRA is involved in maintaining healthy biological flora in the gut. DRA-deficient mice exhibit dysbiosis, especially in butyrate-producing bacterial [20]. Butyrate regulates the assembly and expression of TJ proteins, promotes intestinal barrier function, stabilizes the transcription factor hypoxia inducible factor-1 (HIF-1), and enhances epithelial barrier function [34, 35]. Therefore, abundant expression of DRA in the colonic epithelium may be an indispensable factor for maintaining the integrity of the epithelium, helping to protect the intestinal barrier from damage. In general, overexpression of DRA is beneficial in an inflammatory environment. Although the signaling pathway remains unclear, these findings provide a new research direction.

### Dysfunction in Cl^−^/HCO_3_^−^ exchangers results in the development of mucosal diseases

#### Downregulation of AE2 is closely related to gastric cancer (GC) and hypergastrinemia

AE2 has been found to be downregulated in human GC tissues. It was reported that the occurrence of GC and insufficient gastric acid secretion in GC patients is related to downregulation of AE2 [36]. One of its mechanisms may be that overexpression of AE1/P16 in GC cells promotes degradation of AE2 in GC cells. Under physiological conditions, AE2 mRNA is translated into protein, though translation of AE1 and P16 mRNAs is often inhibited for many reasons. However, the opposite occurs under pathological conditions [37]. After gastric acid secretion is reduced, negative feedback often causes an increase in gastrin. Gastrin is a GI hormone that is mainly produced and secreted by G cells, stimulating gastric acid secretion and gastric fundus mucosa growth [38]. Studies have found that AE2 (a, b) knockout in mice causes G cell proliferation and hypergastrinemia [27]. Gastrin increases expression of AE2 in GC through early growth response 1 (EGR1), but this does not directly affect AE2 [39]. In addition, trastuzumab combined with gastrin has been proven to be effective in treating GC, and one of its mechanisms is upregulation of AE2 in GC tissues [40]. Despite few studies on AE2 in gastric mucosal diseases, AE2 is reportedly a vital membrane protein for preventing GC, and it is expected to become a new target for GC treatment. Indeed, upregulating AE2 expression may improve therapy for GC.

#### DRA participates in the occurrence of IBD and promotes the transition to tumors

Mutations in the SLC26A3 gene can cause congenital chloride diarrhea (CLD) [41]. CLD patients are prone to IBD, including acute and chronic intestinal inflammation. The incidence of IBD in CLD patients is higher than that in healthy individuals, as verified in the latest research [42]. A large amount of evidence shows that IBD is related to intestinal barrier damage [43–45]. Ulcerative colitis (UC) is a type of IBD. Early events in the pathogenesis of UC include structural weakness of the colonic mucosal barrier [46]. DRA is reduced in UC patients, especially in the absence of active inflammation [47]. Ding et al. showed that TNF-α interacts with DRA and downregulate its expression, leading to intestinal inflammation [48]. One possible regulatory mechanism is that TNF-α activates nuclear factor kappa-B (NF-κB), causing it to bind to the DRA promoter, which is also the primary mechanism for downregulating DRA expression [49]. Therefore, anti-TNF-α monoclonal antibodies are commonly used in the clinical treatment of IBD [50]. DRA is also regarded as a colon tumor suppressor [51], and its downregulation is associated with colorectal cancer (CRC) progression [52]. In summary, the absence of DRA may lead to mucosal diseases, including IBD and IBD-related tumors, indicating that DRA may be a new treatment target for these mucosal diseases.

## Cl^−^ channels

Chloride channels are proteins on the cell membrane that are permeable to chloride ions or other anions and are divided into the voltage-gated ClC family, PKA-activated cystic fibrosis transmembrane regulator (CFTR) and intracellular CLICs. Both ClC-2 and ClC-3 belong to the ClC family [53]. As an active substance that protects the mucosa, prostaglandin (PG) has long been reported to stimulate chloride secretion [54]. Similarly, chloride is considered to be key to PG-induced recovery of the mucosal barrier in early stages [55], suggesting a particular relationship between chloride channels and the GI mucosa.

### Expression pattern and functional role of Cl^−^ channels in the GI tract

#### ClC family in the GI tract

Both ClC-2 and ClC-3 belong to the ClC family. The former acts as a voltage-gated anion channel on the plasma membrane of mammalian cells, and the latter mediates exchange of Cl^−^ and H^+^ but is not a voltage-dependent anion channel [53].

ClC-2 is an extensive Cl^−^ channel. In the GI tract, the intestine shows a higher level of ClC-2, whereas the channel is relatively less abundant in the stomach [56]. The expression of ClC-2 protein cannot be detected in isolated rabbit gastric glands, and it is believed that ClC-2 may not be the Cl^−^ transporter secreted by gastric acid in parietal cells [57]. Nevertheless, some researchers have found that ClC-2 protein is expressed on the apical membrane of rabbit gastric parietal cells, with localization similar to that of H^+^/K^+^-ATPase. Loss of ClC-2 can cause a decrease in the number of parietal cells and H^+^/K^+^-ATPase expression, resulting in reduced acid secretion [58, 59]. ClC-2 is highly expressed on the basolateral and TJs of intestinal epithelial cells [60, 61], and its expression level in the early distal colon is higher than that in the late distal colon. Colonic electrical neutral absorption of NaCl and KCl requires basolateral ClC-2 channels [62]; it is also essential for the barrier function of the intestinal epithelium [63–65].

ClC-3 is a strongly outwardly rectifying, electrogenic 2Cl^−^/H^+^ exchanger [66] that is mainly expressed on intracellular vesicles [67, 68]. ClC-3 is also expressed in the ileum and colon [69] and plays a role in regulating cell volume, the cell cycle, apoptosis, and cell migration [70–73].

#### CFTR in the GI tract

CFTR mediates the passive transport of Cl^−^/HCO_3_^−^ [74]; it is expressed in the apical cell membrane of epithelial cells that secrete chloride [75] and participates in regulating the secretion and absorption of various epithelial tissues [76]. The expression of CFTR in the stomach is low [77]. Nevertheless, it has been shown to participate in the secretion of alkaline solid fluid in the “stomach sulcus” [78], and it has a regulatory role in gastric acid secretion [79]. CFTR modulates the cell cycle in GC cells. One of the relevant mechanisms is that CFTR is regulated by AMP-activated protein kinase (AMPK) to change the membrane potential [75]. These findings indicate the importance of CFTR in maintaining the integrity of the gastric mucosal barrier, gastric acid secretion, and the cell cycle. Mutations in CFTR can also lead to impaired mucus hydration and clearance [80]. Secreted bicarbonate is essential to promote mucus regular spreading and hydration [81], which helps to maintain the intestinal flora and bicarbonate barrier [13, 82]. It is worth noting that bicarbonate contributes to relieving GI complications in patients with cystic fibrosis. The drug ivacaftor increases the pH of the proximal small intestine, which may enhance CFTR-mediated bicarbonate secretion [83].

### Dysfunction in Cl^−^ channels results in the development of mucosal diseases

#### ClC family is related to dysfunction of the GI mucosa and GI cancer

In line with the effect of ClC-2 on the mucosal barrier, researchers have found that the gastric mucosa of ClC-2-deficient mice display obvious pathological conditions, such as gastric gland dilatation, reduced gastric gland height, and cell layer disorder [58]. Acid damage is key to gastric ulcers. In an acid injury model, the ClC-2 agonist SPI-8811 enhances the mucosal barrier by increasing the TJ protein occludin in the gastric mucosa, and ZnCl_2_ acts as an inhibitor to weaken this effect [84]. PGE_2_ stimulates the recovery of ischemic ileum mucosa through Cl^−^ secretion mediated by ClC-2 and decreased paracellular permeability [55]. Lubiprostone, a ClC-2 agonist, is used to treat ischemic intestinal injury, redistribute occludin from the cytoplasm to the outer cell membrane, and restore intestinal mucosal barrier function [85]. Regarding the recovery mechanism, ClC-2 regulates caveolin-1 and caveolae-mediated occludin transport and enhances TJ barrier function [63]. Nighot et al. also proposed that ClC-2 acts as a protective factor in colitis [64], but later work showed that ClC-2 reduced the barrier function of the normal mucosa [86]. Overall, ClC-2 has a regulatory effect on the homeostasis and tumorigenicity of adherens junctions (AJs) in the intestinal mucosal epithelium. First, loss of ClC-2 along with disruption of AJs upregulates T cell factor/lymphoid enhancer factor (TCF/LEF1) target genes to promote colitis-associated tumorigenicity. Second, inflammation is promoted in a tumor through reduced colonic crypt differentiation [65].

ClC-3 is highly expressed in GI cancers. Indeed, ClC-3 is regarded as a sign of poor prognosis in GC, and high expression of ClC-3 is significantly related to tumor aggressiveness, lymph node metastasis, and overall survival of patients with GC. ClC-3 is regulated by X-ray repair cross-complementing 5(XRCC5), which binds to its promoter, inducing cancer cell proliferation, migration, and invasion through the transforming growth factor-β (TGF-β)/Smad signaling pathway. Researchers have found that knockdown of ClC-3 inhibits tumor cell proliferation and migration through the phosphatidylinositol 3 kinase (PI3K)/Akt signaling pathway [87, 88]; the Wnt/β-catenin signaling pathway also promotes the occurrence and metastasis of CRC [89]. ClC-3 is highly expressed in neuroendocrine colon cancer [90]. Platelet-activating factor (PAF), a crucial mediator of the pathogenesis of IBD [91], induces activation of ClC-3 in intestinal epithelial cells, thereby causing intracellular acidosis and apoptosis [92]. However, some researchers have reported that expression of ClC-3 is downregulated in IBD patients, which promotes intestinal epithelial cell apoptosis through the mitochondrial pathway, reduces the number of Paneth cells, and weakens expression of antimicrobial peptides to promote bacterial invasion of the mucosa [69]. In addition, ClC-3-mediated regulation of intestinal tissue integrity is worthy of attention. In a lipopolysaccharide (LPS)-induced endotoxemia model, Huang et al. showed that Bax and caspase 3 were significantly increased in the intestinal tissue of mice lacking ClC-3, promoting intestinal cell apoptosis and impairing intestinal integrity [93]. It has also been reported that ClC-3 inhibits the inflammatory response induced by LPS by inhibiting the Toll-like receptor 4 (TLR4)/NF-κB pathway in vivo and in vitro. These results provide a new perspective for inhibiting inflammation based on Cl^−^ channels [94].

#### Dysfunction of CFTR leads to cystic fibrosis (CF)-related mucosal injury and GI cancer

CFTR protein dysfunction directly leads to the clinical symptoms and signs of cystic fibrosis (CF). Studies have shown that patients with CF have a significantly higher risk of GI cancer than the general population [95]. Spasmolytic polypeptide-expressing metaplasia (SPEM) and intestinal metaplasia (IM) are regarded as crucial factors in the occurrence of GC. CFTR was identified as a biomarker of SPEM with inflammation in mice and IM in humans [96]. CFTR is significantly elevated in the serum of patients with GC. Researchers have proposed that serum CFTR be regarded as a new biomarker for GC diagnosis. In addition, CFTR is significantly related to age in GC, and its expression increases with age. Logistic regression analysis confirms that serum CFTR can independently predict the occurrence of GC [97]. The gastric phenotype of CF animals includes submucosal edema and gastric gland mucosal expansion as the most common findings, and mucosal ulcers accompanied by inflammation and erosion are occasionally observed [98]. These results provide a new idea for the future treatment of gastric disease caused by CFTR dysfunction.

It has been reported that compared with the general population, the risk of CRC in adults with CF is 5–10 times higher [99], and CFTR has been identified as a candidate driver gene for CRC. The lack of CFTR promotes tumorigenesis through long-term chronic inflammation caused by an immune response and microbial imbalance [100]. CFTR is significantly downregulated in CRC tissues, and its low expression is related to poor prognosis in CRC patients [101]. Nonfunctional CFTR can lead to bicarbonate deficiency, resulting in decreased and dense intestinal mucus secretion in the CF mouse model [98, 102, 103]. Moreover, intestinal flora imbalance and bacterial overgrowth occur in the development of CF [104–106]. These conditions can directly lead to meconium intestinal obstruction and intestinal inflammation.

## Aquaporins

Aquaporins (AQPs) are a group of endogenous hydrophobic membrane channel proteins on the cell membrane; 13 isoforms (AQP0–AQP12) are involved in the transport and circulation of essential biomolecules. Aquaporins are divided into three subfamilies: orthodox/classic aquaporins, which only transport water; aquaporins that transport small solutes with water; and unorthodox/superaquaporins, which are permeable to charged and uncharged solutes. AQP3 and AQP4 are highly distributed in the stomach; AQP3 and AQP8 are the main subtypes in the colon [107, 108].

### Expression pattern and functional role of AQPs in the GI tract

AQP3 is mainly distributed on the basolateral membrane of gastric and intestinal epithelial cells [109] and is regulated by trefoil factor (TFF) peptides and H_2_O_2_ to participate in cell proliferation and migration [110–114]. AQP3 is also an important contributor to maintaining the mucosal barrier. Knockdown of AQP3 leads to a significant decrease in expression of intestinal epithelial TJ-related proteins claudin-1 and occludin, promoting paracellular permeability and bacterial translocation [18].

Although AQP4 is strongly expressed at the basolateral membrane of gastric parietal cells [115, 116], Wang et al. found that AQP4 does not contribute to gastric fluid secretion, gastric pH, or fasting serum gastrin levels [115]. Nonetheless, AQP4 is essential for the repair of gastric mucosal integrity [117] and is closely related to the degree of parietal cell regeneration [118]. Omeprazole increases parietal cell proliferation and promotes re-epithelialization by upregulating expression of AQP4 [119]. In contrast, the protective effect of calcitonin-related gene peptide on gastric mucosal injury after cerebral ischemia–reperfusion in rats is mediated by inhibiting expression of AQP4 and degranulation of mast cells [120]. Results to date are contradictory and need to be resolved.

AQP8 is located on the apical membrane of colonic epithelial cells [109] and is regarded as a sign of normal colonic epithelial cell proliferation [121]. AQP8 plays an essential role in absorbing intestinal water and may also be involved in intracellular osmotic regulation and mucus flux [122].

### Dysfunction in AQPs results in the development of mucosal diseases

#### Gastric fundic gland polyps (FGPs), precancerous lesions and GC are associated with AQPs

Expression of AQP3 in GC tissues is much higher than that in normal gastric tissues [116]. AQP3 affects the occurrence and development of GC. *Helicobacter pylori* infection is considered to initiate chronic gastritis and GC. In fact, in the presence of *H. pylori*, expression of AQP3 is upregulated through activation of the reactive oxygen species (ROS)-HIF-1α-AQP3-ROS loop, which ultimately leads to GC [123]. As a critical point in precancerous GC, IM is receiving increasing attention. Researchers have proposed that AQP3 is closely related to the severity and classification of IM, and AQP3 can be used as a biomarker of precancerous lesions [124]. In addition, AQP3 promotes the stem cell-like properties of GC by activating the Wnt/glycogen synthase kinase-3β (GSK-3β)/β-catenin signaling pathway [125]. Owing to its transport properties, AQP3 promotes the proliferation of GC cells through the production of energy and lipids [126]. AQP3 also contributes to occurrence of the epithelial–mesenchymal transition (EMT) in GC, which may involve PI3K/Akt/Snail pathway participation [127]. AQP3 correlates positively with lymph node metastasis, low histological classification, and lymphatic vascular invasion [116]. However, some researchers believe that high levels of AQP3 expression are associated with better overall survival [128].

AQP4 becomes rearranged or downregulated in a state of inflammation caused by histamine [129]. When H2 receptor gene knockout mice are infected with *H. pylori*, the ratio of AQP4 to H^+^/K^+^-ATPase expression decreases, and a large amount of SPEM appears [130]. We hypothesize that upregulation of AQP4 might reverse the damage caused by inflammation. Regardless, long-term use of PPIs can lead to the development of sporadic FGP. One of the reasons may be that PPIs upregulate expression of AQP4 and increase the number of parietal cells, resulting in an imbalance of water flow [131, 132]. Nevertheless, the expression level of AQP4 in GC tissues is also significantly lower than that in normal gastric tissues [133], and high expression is associated with poor overall survival [128].

#### IBD and CRC are associated with AQPs

In the early stages of IBD, expression of AQP3 mRNA in the intestinal mucosa is reduced [108]. Decreased intestinal crypt cell proliferation and epithelial cell death and a significant decrease in glycerol permeability are all observed in the AQP3 deletion model. Glycerol treatment significantly increases the survival rate of AQP3-deficient mice and reduces the severity of colitis [134, 135]. Overall, AQP3 can be considered a serum marker of CRC. CRC tissue can release AQP3 and cause an increase in AQP3 content in serum, which is related to CRC differentiation, staging, and survival [136]. Overexpression of AQP3 promotes the migration of CRC cells. Thus, AQP3 may be considered a potential indicator and therapeutic target for colon tumor metastasis and prognosis. AQP3 is highly expressed in CRC tissues and is related to tumor differentiation, lymph nodes, and distant metastasis [137, 138].

The level of AQP8 is significantly reduced in the inflamed colon, with localization changing from an apical to a basolateral position. A reduction in AQP8 has also been confirmed in some chemically induced colitis models [139, 140]. Early studies showed that upregulation of peroxisome proliferator-activated receptor-γ (PPAR-γ) significantly reduces the inflammatory response in IBD mice [141]. For example, the ligand rosiglitazone delayed IBD in interleukin-10-deficient mice and significantly increased expression of the AQP8 gene during the differentiation of surface epithelial cells [142]. It seems reasonable that an increase in AQP8 may benefit IBD repair. However, Zahn et al. found that upregulation of AQP8 mRNA may lead to dehydration of the mucus layer and an increase in adhesion viscosity, which in turn affects mucus adhesion and ultimately disrupts the mucosal barrier of UC patients, especially in the actively inflamed colon [11]. Similar to inflammation, AQP8 is downregulated in CRC tissue. AQP8 can inhibit the growth of tumor cells, and CRC patients with high levels of AQP8 have a better survival time [143, 144].

## Na^+^/H^+^ exchangers

Na^+^/H^+^ exchangers are present on the plasma membranes of all living cells and exchange intracellular H^+^ and extracellular Na^+^ at a ratio of 1:1 to adjust the dynamic balance of intracellular pH and affect cell movement. The NHE family is divided into nine types: NHE1–9 (SLC9A1–9). Except for NHE5, all NHE subtypes have been detected in the GI tract and exhibit segmental differences and distinct cellular localization [145, 146].

### Expression pattern and functional role of Na^+^/H^+^ exchangers in the GI tract

NHE1 is expressed at the basolateral membrane of GI tract epithelial cells [147], and can promote the proliferation of gastric fibroblasts under the induction of insulin-like growth factor II [148]. It has been reported that activating NHE1 increases the migration rate of gastric mucosal epithelial cells but that activating NHE2 under the same conditions leads to the opposite result. A possible explanation is that basolateral membrane proteins, including NHE1, translocate to the leading edge during migration and that apical proteins may stay diffusely in the membrane [149].

NHE2 is expressed on the basolateral membrane of epithelial cells [147, 150], but some researchers report apical membrane expression. NHE2 acts as a downstream effector of TFF proteins, which promote repair of the gastric epithelium [151, 152]. NHE2 is also involved in the mucosal healing of gastric ulcers [153]. PG-induced NHE2 expression and activity inhibition stimulate recovery of the ischemic intestinal barrier [154]. However, deletion of NHE2 impairs barrier recovery, disrupts localization of the TJ proteins occludin and claudin-1 and downregulates their phosphoserine levels. TJ protein phosphorylation is a key step in TJ assembly and is related to the intestinal barrier [19, 155].

NHE8 is located on the apical membrane of GI epithelial cells, especially in the colon, though low levels of mRNA are also detected in the fundus of the stomach. A lack of NHE8 does not affect primary gastric acid secretion, but the pH value of the gastric mucosal surface decreases. It is speculated that NHE8 is indirectly involved in the secretion of gastric bicarbonate [156], which helps to maintain the integrity of the bicarbonate barrier. Similar to what occurs in the stomach, NHE8 is essential for the secretion of intestinal bicarbonate, production of antimicrobial peptides, and synthesis of Muc2 by Paneth cells [12, 15, 157]. In general, Muc2 is a crucial structural component of the mucus layer, and its downregulation allows bacteria to contact the epithelium, directly triggering an inflammatory response [158–160].

### Dysfunction of Na^+^/H^+^ exchangers results in the development of mucosal diseases

#### NHEs participate in acid damage disease and GC

Although activating NHE1 can promote wound healing [149], recent studies have shown that NHE1 is closely related to the occurrence and development of GC. NHE1 enhances the resistance of GC cells to 5-fluorouracil (5-Fu) by regulating the Janus kinase (JAK)/signal transducer and activator of transcription (STAT3) pathway [161]. The NHE1 antisense gene is significant for inhibiting the malignant behavior of human GC cells and growth as well as inducing cell apoptosis [162]. NHE2 gene deletion can lead to a severe gastric phenotype, including the gradual loss of parietal cells and principal cells and the development of gastritis. Mice lacking the NHE2 gene develop glandular mucosal gastritis as early as the 10th day after birth, with a maximum inflammation intensity within 17 to 19 days, followed by total atrophy after one year [14, 16]. Nevertheless, downregulation of NHE2 is still observed for a long time in the regenerating epithelium formed by visual healing ulcers, which affects the gradient of Na^+^ and H^+^ in the cell; this may partly explain recurrence of peptic ulcers [153, 163]. The absence of NHE8 also dramatically promotes gastric ulcer development due to impaired bicarbonate secretion [156].

#### NHEs are involved in the occurrence of ulcers, intestinal inflammation and tumors

Nonsteroidal anti-inflammatory drugs (NSAIDs) are considered to be one of the fundamental causes of peptic ulcers. These drugs may enhance NHE2 proton excretion in colon tissues and play a role in acidification of the colon cavity, which will promote the development of ulcers [164]. Downregulation of NHE2 is closely related to IBD and related symptoms [165]. Inflammatory factors such as TNF-α and interferon-γ (IFN-γ) may inhibit NHE2 expression and activity in intestinal epithelial cells by activating NF-κB [166, 167] though some researchers believe that NHE2 is not altered in the inflamed colon [168]. Furthermore, deletion of NHE8 both causes ulcerative colitis-like disease [169] and promotes the occurrence of colitis-related cancers in mice by increasing expression of leucine-rich repeat containing G protein-coupled receptor 5 (Lgr5) [170]. Somatostatin (SST) can improve the diarrhea symptoms of colitis by increasing expression of NHE8 in the intestine [171], which may involve the Erk1/2-mitogen-activated protein kinase (MAPK) and SSTR2-p38-MAPK pathways [172, 173].

## K^+^ channels

K^+^ channels are the largest ion channel family in mammals and among the transporters first thought to play a role in cell migration. There are four subtypes of K^+^ channels: calcium-activated K^+^ (K_Ca_) channels, internal rectifier K^+^ (K_ir_) channels, voltage-gated K^+^ (K_V_) channels, and double-hole K^+^ (K_2P_) channels. In the intact polarized epithelium, K^+^ channels localize to the root tip or basolateral side. GI ulcers are closely associated with potassium channels, though the opening of different potassium channels can produce different results [174].

### Expression pattern and functional role of K^+^ channels in the GI tract

The K_ATP_ channel, which is activated by ATP, is found in vascular endothelial cells [175]. There is much evidence that activating K_ATP_ channels increases gastric mucosal blood flow in the gastric epithelial barrier and promotes mucosal repair. For example, in treatment of gastric ulcers, endogenous vasodilator calcitonin gene-related peptide and irsogladine maleate partially activate K_ATP_ channels, increase gastric mucosal blood flow during gastric acid challenge, and mediate gastric protection [21, 176]. In addition, the CO released by the tricarbonyldichlororuthenium (II) dimer can prevent the gastric mucosal oxidative damage caused by changes in gastric blood flow due to ischemia/reperfusion involving the activity of K_ATP_ channels [177].

Recirculation of K^+^ on the mucosal side of parietal cells is required for gastric acid secretion [178]. KCNQ1, also known as K_V_7.1, is a voltage-dependent K channel that regulates gastric acid secretion [179], and its expression is increased by gastrin [180]. KCNQ1 is located in tubulovesicles and the apical membrane of parietal cells [181]. Acid secretion by parietal cells requires potassium channels and functional H^+^–K^+^-ATPase and potassium channels. Potassium secretion is necessary to maintain continuous H^+^–K^+^-ATPase activity, and KCNQ1 is the main apical potassium channel [182].

KCNN4, a medium-conductivity Ca^2+^-dependent K^+^ (IK) channel localizing to the apical and basolateral membrane of intestinal cells, is involved in duodenal bicarbonate and colonic Cl- secretion [183–185]. Early research found that KCNN4 causes the α-defensin secreted by Paneth cells in the small intestine to respond to bacteria and has mucosal defense effects that kill bacterial pathogens [186]. In terms of immunity, KCNN4 has a regulatory effect on T cell activation [187, 188] and participates in recruitment of monocytes, macrophages, and possibly natural killer cells to the site of inflammation [189].

### Dysfunction of K^+^ channels result in the development of mucosal diseases

#### Downregulation or absence of different K^+^ channels promotes the occurrence of gastric ulcers and gastric tumors

Early studies have shown that using the K_ATP_ channel opener diazoxide can significantly reduce acute gastric injury or gastric ulcer in rats caused by indomethacin or ethanol, thereby accelerating mucosal repair. Conversely, the K_ATP_ channel inhibitor glibenclamide enhances damage and weakens the protective effect of H_2_S on gastric mucosal injury [190–193]. NSAIDs have been shown to stimulate K^+^ efflux and increase cell membrane permeability [194], which may be related to peptic ulcers caused by NSAIDs. As early as the 1960s, potassium ions themselves were shown to be the cause of certain types of peptic ulcers [195–197] though it remains unclear whether this is the result of K_ATP_ channel participation. In general, the role of K_ATP_ channels in repair needs to be confirmed.

KCNQ1 gene mutations have long been proposed to be related to increased susceptibility to dysplasia and premalignant adenomatous hyperplasia of the stomach [198]. KCNQ1 gene polymorphism may also have predictive or prognostic value in determining susceptibility, risk, and survival in Chinese patients with GC [199]. Studies have shown that loss of KCNQ1 is likely to lead to the development of pyloric tumors [200] and that KCNQ1 is involved in the proliferation of GC cells regulated by atrial natriuretic peptide [201]. Such evidence confirms the role of KCNQ1 in the occurrence and development of GC, and KCNQ1 may become a target for the treatment of GC.

### KCNN4 is closely related to IBD, CRC and tumor resistance

KCNN4 expression and activity in the colon of patients with active UC are significantly reduced. This change is considered a possible cause of diarrhea in these patients [202]. In addition, inhibition of KCNN4 causes T cell receptors to stimulate Ca^2+^ influx and affects T lymphocyte Ca^2+^ signal transduction, which is conducive to relieving T cell-mediated colitis [203]. A recent study reported that a pharmacological KCNN4 channel opener can stabilize intestinal epithelial barrier function in vitro [204]. Additionally, data from a study in Australia showed that the level of KCNN4 mRNA in patients with NOD2 gene mutations was significantly reduced, leading to Paneth cell defense defects and the development of CD [205]. For IBD patients, upregulating expression of KCNN4 may constitute a future treatment strategy. Compared with normal tissues, KCNN4 is upregulated in CRC tissues, which may be an essential factor in the occurrence and progression of CRC [206]. KCNN4 is also upregulated by phosphatase of regenerating liver-3 (PRL-3) and participates in PRL-3-induced EMT via the calcium/CaM-kinase II/GSK-3 β pathway [207]; KCNN4 is also significantly related to the treatment of CRC resistance. Drug-resistant cells express more KCNN4 than cisplatin-sensitive cells, promoting cisplatin absorption in the former and increasing their apoptosis [208].

## Discussion

Previous studies have revealed the role of partial ion channels and transporters in the repair of the GI mucosa and provided convincing evidence that these channels and transporters promote the proliferation and migration of adjacent cells, stabilize the structure of AJs and TJs, protect the mucous barrier, and increase mucosal blood flow. These functions are likely to make ion channels and transporters therapeutic targets for treating inflammation and even cancer. This review provides a basic and systemic summary of the field, which will prompt researchers to focus on the functional diversity of ion transporters in GI mucosal diseases, providing a novel perspective not only for therapy but also, more importantly, for prevention.

## References

[1] Laine L, Takeuchi K, Tarnawski A (2008). Gastric mucosal defense and cytoprotection: bench to bedside. Gastroenterology.

[2] McColl KE (2012). The elegance of the gastric mucosal barrier: designed by nature for nature. Gut.

[3] Camilleri M (2019). Leaky gut: mechanisms, measurement and clinical implications in humans. Gut.

[4] Schubert ML, Peura DA (2008). Control of gastric acid secretion in health and disease. Gastroenterology.

[5] Conti L, Annibale B, Lahner E (2020). Autoimmune gastritis and gastric microbiota. Microorganisms.

[6] Khor B, Gardet A, Xavier RJ (2011). Genetics and pathogenesis of inflammatory bowel disease. Nature.

[7] Natividad JM, Verdu EF (2013). Modulation of intestinal barrier by intestinal microbiota: pathological and therapeutic implications. Pharmacol Res.

[8] Levin LR, Buck J (2015). Physiological roles of acid-base sensors. Annu Rev Physiol.

[9] Lanas A, Chan FKL (2017). Peptic ulcer disease. Lancet.

[10] Xiao F, Yu Q, Li J, Johansson ME, Singh AK, Xia W, Riederer B, Engelhardt R, Montrose M, Soleimani M, Tian DA, Xu G, Hansson GC, Seidler U (2014). Slc26a3 deficiency is associated with loss of colonic HCO3 (-) secretion, absence of a firm mucus layer and barrier impairment in mice. Acta Physiol (Oxf).

[11] Zahn A, Moehle C, Langmann T, Ehehalt R, Autschbach F, Stremmel W, Schmitz G (2007). Aquaporin-8 expression is reduced in ileum and induced in colon of patients with ulcerative colitis. World J Gastroenterol.

[12] Xu H, Zhang B, Li J, Wang C, Chen H, Ghishan FK (2012). Impaired mucin synthesis and bicarbonate secretion in the colon of NHE8 knockout mice. Am J Physiol Gastrointest Liver Physiol.

[13] Singh AK, Sjoblom M, Zheng W, Krabbenhoft A, Riederer B, Rausch B, Manns MP, Soleimani M, Seidler U (2008). CFTR and its key role in in vivo resting and luminal acid-induced duodenal HCO3- secretion. Acta Physiol (Oxf).

[14] Schultheis PJ, Clarke LL, Meneton P, Harline M, Boivin GP, Stemmermann G, Duffy JJ, Doetschman T, Miller ML, Shull GE (1998). Targeted disruption of the murine Na+/H+ exchanger isoform 2 gene causes reduced viability of gastric parietal cells and loss of net acid secretion. J Clin Invest.

[15] Wang A, Li J, Zhao Y, Johansson ME, Xu H, Ghishan FK (2015). Loss of NHE8 expression impairs intestinal mucosal integrity. Am J Physiol Gastrointest Liver Physiol.

[16] Boivin GP, Schultheis PJ, Shull GE, Stemmermann GN (2000). Variant form of diffuse corporal gastritis in NHE2 knockout mice. Comp Med.

[17] Ding X, Li D, Li M, Wang H, He Q, Wang Y, Yu H, Tian D, Yu Q (2018). SLC26A3 (DRA) prevents TNF-alpha-induced barrier dysfunction and dextran sulfate sodium-induced acute colitis. Lab Invest.

[18] Zhang W, Xu Y, Chen Z, Xu Z, Xu H (2011). Knockdown of aquaporin 3 is involved in intestinal barrier integrity impairment. FEBS Lett.

[19] Moeser AJ, Nighot PK, Ryan KA, Simpson JE, Clarke LL, Blikslager AT (2008). Mice lacking the Na+/H+ exchanger 2 have impaired recovery of intestinal barrier function. Am J Physiol Gastrointest Liver Physiol.

[20] Kumar A, Priyamvada S, Ge Y, Jayawardena D, Singhal M, Anbazhagan AN, Chatterjee I, Dayal A, Patel M, Zadeh K, Saksena S, Alrefai WA, Gill RK, Zadeh M, Zhao N, Mohamadzadeh M, Dudeja PK (2021). A novel role of SLC26A3 in the maintenance of intestinal epithelial barrier integrity. Gastroenterology.

[21] Doi K, Nagao T, Kawakubo K, Ibayashi S, Aoyagi K, Yano Y, Yamamoto C, Kanamoto K, Iida M, Sadoshima S, Fujishima M (1998). Calcitonin gene-related peptide affords gastric mucosal protection by activating potassium channel in Wistar rat. Gastroenterology.

[22] Romero MF, Chen AP, Parker MD, Boron WF (2013). The SLC4 family of bicarbonate (HCO(3)(-)) transporters. Mol Aspects Med.

[23] Seidler U, Nikolovska K (2019). Slc26 family of anion transporters in the gastrointestinal tract: expression, function, regulation, and role in disease. Compr Physiol.

[24] Mount DB, Romero MF (2004). The SLC26 gene family of multifunctional anion exchangers. Pflugers Arch.

[25] El Khouri E, Toure A (2014). Functional interaction of the cystic fibrosis transmembrane conductance regulator with members of the SLC26 family of anion transporters (SLC26A8 and SLC26A9): physiological and pathophysiological relevance. Int J Biochem Cell Biol.

[26] McDaniel N, Lytle C (1999). Parietal cells express high levels of Na-K-2Cl cotransporter on migrating into the gastric gland neck. Am J Physiol.

[27] Recalde S, Muruzabal F, Looije N, Kunne C, Burrell MA, Saez E, Martinez-Anso E, Salas JT, Mardones P, Prieto J, Medina JF, Elferink RP (2006). Inefficient chronic activation of parietal cells in Ae2a, b(-/-) mice. Am J Pathol.

[28] Rossmann H, Bachmann O, Wang Z, Shull GE, Obermaier B, Stuart-Tilley A, Alper SL, Seidler U (2001). Differential expression and regulation of AE2 anion exchanger subtypes in rabbit parietal and mucous cells. J Physiol.

[29] Gawenis LR, Ledoussal C, Judd LM, Prasad V, Alper SL, Stuart-Tilley A, Woo AL, Grisham C, Sanford LP, Doetschman T, Miller ML, Shull GE (2004). Mice with a targeted disruption of the AE2 Cl-/HCO3- exchanger are achlorhydric. J Biol Chem.

[30] Humphreys BD, Chernova MN, Jiang L, Zhang Y, Alper SL (1997). NH4Cl activates AE2 anion exchanger in Xenopus oocytes at acidic pHi. Am J Physiol.

[31] Morgan PE, Pastorekova S, Stuart-Tilley AK, Alper SL, Casey JR (2007). Interactions of transmembrane carbonic anhydrase, CAIX, with bicarbonate transporters. Am J Physiol Cell Physiol.

[32] Svastova E, Witarski W, Csaderova L, Kosik I, Skvarkova L, Hulikova A, Zatovicova M, Barathova M, Kopacek J, Pastorek J, Pastorekova S (2012). Carbonic anhydrase IX interacts with bicarbonate transporters in lamellipodia and increases cell migration via its catalytic domain. J Biol Chem.

[33] Xiao F, Juric M, Li J, Riederer B, Yeruva S, Singh AK, Zheng L, Glage S, Kollias G, Dudeja P, Tian DA, Xu G, Zhu J, Bachmann O, Seidler U (2012). Loss of downregulated in adenoma (DRA) impairs mucosal HCO3(-) secretion in murine ileocolonic inflammation. Inflamm Bowel Dis.

[34] Wang HB, Wang PY, Wang X, Wan YL, Liu YC (2012). Butyrate enhances intestinal epithelial barrier function via up-regulation of tight junction protein Claudin-1 transcription. Dig Dis Sci.

[35] Kelly CJ, Zheng L, Campbell EL, Saeedi B, Scholz CC, Bayless AJ, Wilson KE, Glover LE, Kominsky DJ, Magnuson A, Weir TL, Ehrentraut SF, Pickel C, Kuhn KA, Lanis JM, Nguyen V, Taylor CT, Colgan SP (2015). Crosstalk between microbiota-derived short-chain fatty acids and intestinal epithelial HIF augments tissue barrier function. Cell Host Microbe.

[36] Yang Y, Wu PP, Wu J, Shen WW, Wu YL, Fu AF, Zheng L, Jin XL, Fu GH (2008). Expression of anion exchanger 2 in human gastric cancer. Exp Oncol.

[37] Wang T, Fei HJ, Yang Y, Jiang XS, Yan M, Zeng Z, Wu J, Song LJ, Tian H, Fu GH (2016). Expression of AE1/p16 promoted degradation of AE2 in gastric cancer cells. BMC Cancer.

[38] Rehfeld JF (2021). Gastrin and the moderate hypergastrinemias. Int J Mol Sci.

[39] Wang T, Zhao L, Yang Y, Tian H, Suo WH, Yan M, Fu GH (2013). EGR1 is critical for gastrin-dependent upregulation of anion exchanger 2 in gastric cancer cells. FEBS J.

[40] Cui Y, Li SB, Peng XC, Wu J, Fu GH (2015). Trastuzumab inhibits growth of HER2-negative gastric cancer cells through gastrin-initialized CCKBR signaling. Dig Dis Sci.

[41] Wedenoja S, Pekansaari E, Hoglund P, Makela S, Holmberg C, Kere J (2011). Update on SLC26A3 mutations in congenital chloride diarrhea. Hum Mutat.

[42] Norsa L, Berni Canani R, Duclaux-Loras R, Bequet E, Koglmeier J, Russell RK, Uhlig HH, Travis S, Hollis J, Koletzko S, Grimaldi G, Castaldo G, Rodrigues A, Deflandre J, Dembinski L, Shah N, Heinz-Erian P, Janecke A, Leskinen S, Wedenoja S, Koskela R, Lachaux A, Kolho KL, Ruemmele FM (2021). Inflammatory bowel disease in patients with congenital chloride diarrhoea. J Crohns Colitis.

[43] Priyamvada S, Gomes R, Gill RK, Saksena S, Alrefai WA, Dudeja PK (2015). Mechanisms underlying dysregulation of electrolyte absorption in inflammatory bowel disease-associated diarrhea. Inflamm Bowel Dis.

[44] Wedenoja S, Hoglund P, Holmberg C (2010). Review article: the clinical management of congenital chloride diarrhoea. Aliment Pharmacol Ther.

[45] Michielan A, D'Inca R (2015). Intestinal permeability in inflammatory bowel disease: pathogenesis, clinical evaluation, and therapy of leaky gut. Mediators Inflamm.

[46] van der Post S, Jabbar KS, Birchenough G, Arike L, Akhtar N, Sjovall H, Johansson MEV, Hansson GC (2019). Structural weakening of the colonic mucus barrier is an early event in ulcerative colitis pathogenesis. Gut.

[47] Farkas K, Yeruva S, Rakonczay Z, Ludolph L, Molnar T, Nagy F, Szepes Z, Schnur A, Wittmann T, Hubricht J, Riederer B, Venglovecz V, Lazar G, Kiraly M, Zsembery A, Varga G, Seidler U, Hegyi P (2011). New therapeutic targets in ulcerative colitis: the importance of ion transporters in the human colon. Inflamm Bowel Dis.

[48] Ding X, Li D, Li M, Tian D, Yu H, Yu Q (2018). Tumor necrosis factor-alpha acts reciprocally with solute carrier family 26, member 3, (downregulated-in-adenoma) and reduces its expression, leading to intestinal inflammation. Int J Mol Med.

[49] Kumar A, Chatterjee I, Gujral T, Alakkam A, Coffing H, Anbazhagan AN, Borthakur A, Saksena S, Gill RK, Alrefai WA, Dudeja PK (2017). Activation of nuclear factor-kappab by tumor necrosis factor in intestinal epithelial cells and mouse intestinal epithelia reduces expression of the chloride transporter SLC26A3. Gastroenterology.

[50] Sandborn WJ, van Assche G, Reinisch W, Colombel JF, D'Haens G, Wolf DC, Kron M, Tighe MB, Lazar A, Thakkar RB (2012). Adalimumab induces and maintains clinical remission in patients with moderate-to-severe ulcerative colitis. Gastroenterology.

[51] Bhutia YD, Babu E, Ramachandran S, Yang S, Thangaraju M, Ganapathy V (2016). SLC transporters as a novel class of tumour suppressors: identity, function and molecular mechanisms. Biochem J.

[52] Antalis TM, Reeder JA, Gotley DC, Byeon MK, Walsh MD, Henderson KW, Papas TS, Schweinfest CW (1998). Down-regulation of the down-regulated in adenoma (DRA) gene correlates with colon tumor progression. Clin Cancer Res.

[53] Duran C, Thompson CH, Xiao Q, Hartzell HC (2010). Chloride channels: often enigmatic, rarely predictable. Annu Rev Physiol.

[54] Bunce KT, Spraggs CF (1988). Stimulation of electrogenic chloride secretion by prostaglandin E2 in guinea-pig isolated gastric mucosa. J Physiol.

[55] Moeser AJ, Haskell MM, Shifflett DE, Little D, Schultz BD, Blikslager AT (2004). ClC-2 chloride secretion mediates prostaglandin-induced recovery of barrier function in ischemia-injured porcine ileum. Gastroenterology.

[56] Thiemann A, Grunder S, Pusch M, Jentsch TJ (1992). A chloride channel widely expressed in epithelial and non-epithelial cells. Nature.

[57] Hori K, Takahashi Y, Horikawa N, Furukawa T, Tsukada K, Takeguchi N, Sakai H (2004). Is the ClC-2 chloride channel involved in the Cl- secretory mechanism of gastric parietal cells?. FEBS Lett.

[58] Nighot MP, Nighot PK, Ma TY, Malinowska DH, Shull GE, Cuppoletti J, Blikslager AT (2015). Genetic ablation of the ClC-2 Cl- channel disrupts mouse gastric parietal cell acid secretion. PLoS ONE.

[59] Sherry AM, Malinowska DH, Morris RE, Ciraolo GM, Cuppoletti J (2001). Localization of ClC-2 Cl- channels in rabbit gastric mucosa. Am J Physiol Cell Physiol.

[60] Lipecka J, Bali M, Thomas A, Fanen P, Edelman A, Fritsch J (2002). Distribution of ClC-2 chloride channel in rat and human epithelial tissues. Am J Physiol Cell Physiol.

[61] Gyomorey K, Yeger H, Ackerley C, Garami E, Bear CE (2000). Expression of the chloride channel ClC-2 in the murine small intestine epithelium. Am J Physiol Cell Physiol.

[62] Catalan MA, Flores CA, Gonzalez-Begne M, Zhang Y, Sepulveda FV, Melvin JE (2012). Severe defects in absorptive ion transport in distal colons of mice that lack ClC-2 channels. Gastroenterology.

[63] Nighot PK, Leung L, Ma TY (2017). Chloride channel ClC- 2 enhances intestinal epithelial tight junction barrier function via regulation of caveolin-1 and caveolar trafficking of occludin. Exp Cell Res.

[64] Nighot P, Young K, Nighot M, Rawat M, Sung EJ, Maharshak N, Plevy SE, Ma T, Blikslager A (2013). Chloride channel ClC-2 is a key factor in the development of DSS-induced murine colitis. Inflamm Bowel Dis.

[65] Jin Y, Ibrahim D, Magness ST, Blikslager AT (2018). Knockout of ClC-2 reveals critical functions of adherens junctions in colonic homeostasis and tumorigenicity. Am J Physiol Gastrointest Liver Physiol.

[66] Guzman RE, Grieschat M, Fahlke C, Alekov AK (2013). ClC-3 is an intracellular chloride/proton exchanger with large voltage-dependent nonlinear capacitance. ACS Chem Neurosci.

[67] Maritzen T, Keating DJ, Neagoe I, Zdebik AA, Jentsch TJ (2008). Role of the vesicular chloride transporter ClC-3 in neuroendocrine tissue. J Neurosci.

[68] Stobrawa SM, Breiderhoff T, Takamori S, Engel D, Schweizer M, Zdebik AA, Bosl MR, Ruether K, Jahn H, Draguhn A, Jahn R, Jentsch TJ (2001). Disruption of ClC-3, a chloride channel expressed on synaptic vesicles, leads to a loss of the hippocampus. Neuron.

[69] Huang LY, He Q, Liang SJ, Su YX, Xiong LX, Wu QQ, Wu QY, Tao J, Wang JP, Tang YB, Lv XF, Liu J, Guan YY, Pang RP, Zhou JG (2014). ClC-3 chloride channel/antiporter defect contributes to inflammatory bowel disease in humans and mice. Gut.

[70] Duan D, Winter C, Cowley S, Hume JR, Horowitz B (1997). Molecular identification of a volume-regulated chloride channel. Nature.

[71] Mao J, Chen L, Xu B, Wang L, Wang W, Li M, Zheng M, Li H, Guo J, Li W, Jacob TJ, Wang L (2009). Volume-activated chloride channels contribute to cell-cycle-dependent regulation of HeLa cell migration. Biochem Pharmacol.

[72] Guan YY, Wang GL, Zhou JG (2006). The ClC-3 Cl- channel in cell volume regulation, proliferation and apoptosis in vascular smooth muscle cells. Trends Pharmacol Sci.

[73] Ganapathi SB, Wei SG, Zaremba A, Lamb FS, Shears SB (2013). Functional regulation of ClC-3 in the migration of vascular smooth muscle cells. Hypertension.

[74] Hwang TC, Kirk KL (2013). The CFTR ion channel: gating, regulation, and anion permeation. Cold Spring Harb Perspect Med.

[75] Zhu L, Yu XJ, Xing S, Jin F, Yang WJ (2018). Involvement of AMP-activated protein kinase (AMPK) in regulation of cell membrane potential in a gastric cancer cell line. Sci Rep.

[76] Saint-Criq V, Gray MA (2017). Role of CFTR in epithelial physiology. Cell Mol Life Sci.

[77] Liu X, Li T, Riederer B, Lenzen H, Ludolph L, Yeruva S, Tuo B, Soleimani M, Seidler U (2015). Loss of Slc26a9 anion transporter alters intestinal electrolyte and HCO3(-) transport and reduces survival in CFTR-deficient mice. Pflugers Arch.

[78] Eberle JA, Muller-Roth KL, Widmayer P, Chubanov V, Gudermann T, Breer H (2013). Putative interaction of brush cells with bicarbonate secreting cells in the proximal corpus mucosa. Front Physiol.

[79] Sidani SM, Kirchhoff P, Socrates T, Stelter L, Ferreira E, Caputo C, Roberts KE, Bell RL, Egan ME, Geibel JP (2007). DeltaF508 mutation results in impaired gastric acid secretion. J Biol Chem.

[80] Shteinberg M, Haq IJ, Polineni D, Davies JC (2021). Cystic fibrosis. Lancet.

[81] Quinton PM (2008). Cystic fibrosis: impaired bicarbonate secretion and mucoviscidosis. Lancet.

[82] Norkina O, Burnett TG, De Lisle RC (2004). Bacterial overgrowth in the cystic fibrosis transmembrane conductance regulator null mouse small intestine. Infect Immun.

[83] Gelfond D, Heltshe S, Ma C, Rowe SM, Frederick C, Uluer A, Sicilian L, Konstan M, Tullis E, Roach RN, Griffin K, Joseloff E, Borowitz D (2017). Impact of CFTR modulation on intestinal pH, motility, and clinical outcomes in patients with cystic fibrosis and the G551D mutation. Clin Transl Gastroenterol.

[84] Nighot M, Moeser A, Ueno R, Blikslager A (2012). Gastro protective properties of the novel prostone SPI-8811 against acid-injured porcine mucosa. World J Gastroenterol.

[85] Moeser AJ, Nighot PK, Engelke KJ, Ueno R, Blikslager AT (2007). Recovery of mucosal barrier function in ischemic porcine ileum and colon is stimulated by a novel agonist of the ClC-2 chloride channel, lubiprostone. Am J Physiol Gastrointest Liver Physiol.

[86] Nighot PK, Blikslager AT (2010). ClC-2 regulates mucosal barrier function associated with structural changes to the villus and epithelial tight junction. Am J Physiol Gastrointest Liver Physiol.

[87] Peng J, Chen W, Chen J, Yuan Y, Zhang J, He Y (2018). Overexpression of chloride channel-3 predicts unfavorable prognosis and promotes cellular invasion in gastric cancer. Cancer Manag Res.

[88] Gu Z, Li Y, Yang X, Yu M, Chen Z, Zhao C, Chen L, Wang L (2018). Overexpression of CLC-3 is regulated by XRCC5 and is a poor prognostic biomarker for gastric cancer. J Hematol Oncol.

[89] Mu H, Mu L, Gao J (2020). Suppression of CLC-3 reduces the proliferation, invasion and migration of colorectal cancer through Wnt/beta-catenin signaling pathway. Biochem Biophys Res Commun.

[90] Weylandt KH, Nebrig M, Jansen-Rosseck N, Amey JS, Carmena D, Wiedenmann B, Higgins CF, Sardini A (2007). ClC-3 expression enhances etoposide resistance by increasing acidification of the late endocytic compartment. Mol Cancer Ther.

[91] Lu J, Caplan MS, Saraf AP, Li D, Adler L, Liu X, Jilling T (2004). Platelet-activating factor-induced apoptosis is blocked by Bcl-2 in rat intestinal epithelial cells. Am J Physiol Gastrointest Liver Physiol.

[92] Claud EC, Lu J, Wang XQ, Abe M, Petrof EO, Sun J, Nelson DJ, Marks J, Jilling T (2008). Platelet-activating factor-induced chloride channel activation is associated with intracellular acidosis and apoptosis of intestinal epithelial cells. Am J Physiol Gastrointest Liver Physiol.

[93] Huang LY, Li YJ, Li PP, Li HC, Ma P (2018). Aggravated intestinal apoptosis by ClC-3 deletion is lethal to mice endotoxemia. Cell Biol Int.

[94] Xiang NL, Liu J, Liao YJ, Huang YW, Wu Z, Bai ZQ, Lin X, Zhang JH (2016). Abrogating ClC-3 inhibits LPS-induced inflammation via blocking the TLR4/NF-kappaB pathway. Sci Rep.

[95] Yamada A, Komaki Y, Komaki F, Micic D, Zullow S, Sakuraba A (2018). Risk of gastrointestinal cancers in patients with cystic fibrosis: a systematic review and meta-analysis. Lancet Oncol.

[96] Weis VG, Sousa JF, LaFleur BJ, Nam KT, Weis JA, Finke PE, Ameen NA, Fox JG, Goldenring JR (2013). Heterogeneity in mouse spasmolytic polypeptide-expressing metaplasia lineages identifies markers of metaplastic progression. Gut.

[97] Liu H, Wu W, Liu Y, Zhang C, Zhou Z (2014). Predictive value of cystic fibrosis transmembrane conductance regulator (CFTR) in the diagnosis of gastric cancer. Clin Invest Med.

[98] Sun X, Olivier AK, Yi Y, Pope CE, Hayden HS, Liang B, Sui H, Zhou W, Hager KR, Zhang Y, Liu X, Yan Z, Fisher JT, Keiser NW, Song Y, Tyler SR, Goeken JA, Kinyon JM, Radey MC, Fligg D, Wang X, Xie W, Lynch TJ, Kaminsky PM, Brittnacher MJ, Miller SI, Parekh K, Meyerholz DK, Hoffman LR, Frana T, Stewart ZA, Engelhardt JF (2014). Gastrointestinal pathology in juvenile and adult CFTR-knockout ferrets. Am J Pathol.

[99] Hadjiliadis D, Khoruts A, Zauber AG, Hempstead SE, Maisonneuve P, Lowenfels AB, Cystic Fibrosis Colorectal Cancer Screening Task, F (2018). Cystic fibrosis colorectal cancer screening consensus recommendations. Gastroenterology.

[100] Than BL, Linnekamp JF, Starr TK, Largaespada DA, Rod A, Zhang Y, Bruner V, Abrahante J, Schumann A, Luczak T, Walter J, Niemczyk A, O'Sullivan MG, Medema JP, Fijneman RJ, Meijer GA, Van den Broek E, Hodges CA, Scott PM, Vermeulen L, Cormier RT (2016). CFTR is a tumor suppressor gene in murine and human intestinal cancer. Oncogene.

[101] Gustafsson JK, Linden SK, Alwan AH, Scholte BJ, Hansson GC, Sjovall H (2015). Carbachol-induced colonic mucus formation requires transport via NKCC1, K(+) channels and CFTR. Pflugers Arch.

[102] Gustafsson JK, Ermund A, Ambort D, Johansson ME, Nilsson HE, Thorell K, Hebert H, Sjovall H, Hansson GC (2012). Bicarbonate and functional CFTR channel are required for proper mucin secretion and link cystic fibrosis with its mucus phenotype. J Exp Med.

[103] Garcia MA, Yang N, Quinton PM (2009). Normal mouse intestinal mucus release requires cystic fibrosis transmembrane regulator-dependent bicarbonate secretion. J Clin Invest.

[104] Hoffman LR, Pope CE, Hayden HS, Heltshe S, Levy R, McNamara S, Jacobs MA, Rohmer L, Radey M, Ramsey BW, Brittnacher MJ, Borenstein E, Miller SI (2014). Escherichia coli dysbiosis correlates with gastrointestinal dysfunction in children with cystic fibrosis. Clin Infect Dis.

[105] Lynch SV, Goldfarb KC, Wild YK, Kong W, De Lisle RC, Brodie EL (2013). Cystic fibrosis transmembrane conductance regulator knockout mice exhibit aberrant gastrointestinal microbiota. Gut Microbes.

[106] Meeker SM, Mears KS, Sangwan N, Brittnacher MJ, Weiss EJ, Treuting PM, Tolley N, Pope CE, Hager KR, Vo AT, Paik J, Frevert CW, Hayden HS, Hoffman LR, Miller SI, Hajjar AM (2020). CFTR dysregulation drives active selection of the gut microbiome. PLoS Pathog.

[107] Sisto M, Ribatti D, Lisi S (2019). Aquaporin water channels: New perspectives on the potential role in inflammation. Adv Protein Chem Struct Biol.

[108] Zhu C, Chen Z, Jiang Z (2016). Expression, distribution and role of aquaporin water channels in human and animal stomach and intestines. Int J Mol Sci.

[109] Zhao GX, Dong PP, Peng R, Li J, Zhang DY, Wang JY, Shen XZ, Dong L, Sun JY (2016). Expression, localization and possible functions of aquaporins 3 and 8 in rat digestive system. Biotech Histochem.

[110] Marchbank T, Playford RJ (2018). Trefoil factor family peptides enhance cell migration by increasing cellular osmotic permeability and aquaporin 3 levels. FASEB J.

[111] Taupin D, Podolsky DK (2003). Trefoil factors: initiators of mucosal healing. Nat Rev Mol Cell Biol.

[112] Huang Y, Zhu Z, Sun M, Wang J, Guo R, Shen L, Wu W (2010). Critical role of aquaporin-3 in the human epidermal growth factor-induced migration and proliferation in the human gastric adenocarcinoma cells. Cancer Biol Ther.

[113] Thiagarajah JR, Chang J, Goettel JA, Verkman AS, Lencer WI (2017). Aquaporin-3 mediates hydrogen peroxide-dependent responses to environmental stress in colonic epithelia. Proc Natl Acad Sci U S A.

[114] van der Vliet A, Janssen-Heininger YM (2014). Hydrogen peroxide as a damage signal in tissue injury and inflammation: murderer, mediator, or messenger?. J Cell Biochem.

[115] Wang KS, Komar AR, Ma T, Filiz F, McLeroy J, Hoda K, Verkman AS, Bastidas JA (2000). Gastric acid secretion in aquaporin-4 knockout mice. Am J Physiol Gastrointest Liver Physiol.

[116] Shen L, Zhu Z, Huang Y, Shu Y, Sun M, Xu H, Zhang G, Guo R, Wei W, Wu W (2010). Expression profile of multiple aquaporins in human gastric carcinoma and its clinical significance. Biomed Pharmacother.

[117] Bodis B, Nagy G, Nemeth P, Mozsik G (2001). Active water selective channels in the stomach: investigation of aquaporins after ethanol and capsaicin treatment in rats. J Physiol Paris.

[118] Matsuoka T, Kobayashi M, Sugimoto T, Araki K (2005). An immunocytochemical study of regeneration of gastric epithelia in rat experimental ulcers. Med Mol Morphol.

[119] Kengkoom K, Tirawanchai NN, Angkhasirisap W, Ampawong S (2017). Omeprazole preserves the RER in chief cells and enhances re-epithelialization of parietal cells with SOD and AQP-4 up-regulation in ethanol-induced gastritis rats. Exp Ther Med.

[120] Feng G, Xu X, Wang Q, Liu Z, Li Z, Liu G (2010). The protective effects of calcitonin gene-related peptide on gastric mucosa injury after cerebral ischemia reperfusion in rats. Regul Pept.

[121] Fischer H, Stenling R, Rubio C, Lindblom A (2001). Differential expression of aquaporin 8 in human colonic epithelial cells and colorectal tumors. BMC Physiol.

[122] Calamita G, Mazzone A, Bizzoca A, Cavalier A, Cassano G, Thomas D, Svelto M (2001). Expression and immunolocalization of the aquaporin-8 water channel in rat gastrointestinal tract. Eur J Cell Biol.

[123] Wen J, Wang Y, Gao C, Zhang G, You Q, Zhang W, Zhang Z, Wang S, Peng G, Shen L (2018). Helicobacter pylori infection promotes Aquaporin 3 expression via the ROS-HIF-1alpha-AQP3-ROS loop in stomach mucosa: a potential novel mechanism for cancer pathogenesis. Oncogene.

[124] Zhao H, Wen J, Dong X, He R, Gao C, Zhang W, Zhang Z, Shen L (2017). Identification of AQP3 and CD24 as biomarkers for carcinogenesis of gastric intestinal metaplasia. Oncotarget.

[125] Zhou Y, Wang Y, Wen J, Zhao H, Dong X, Zhang Z, Wang S, Shen L (2016). Aquaporin 3 promotes the stem-like properties of gastric cancer cells via Wnt/GSK-3beta/beta-catenin pathway. Oncotarget.

[126] Li Z, Li B, Zhang L, Chen L, Sun G, Zhang Q, Wang J, Zhi X, Wang L, Xu Z, Xu H (2016). The proliferation impairment induced by AQP3 deficiency is the result of glycerol uptake and metabolism inhibition in gastric cancer cells. Tumour Biol.

[127] Chen J, Wang T, Zhou YC, Gao F, Zhang ZH, Xu H, Wang SL, Shen LZ (2014). Aquaporin 3 promotes epithelial-mesenchymal transition in gastric cancer. J Exp Clin Cancer Res.

[128] Thapa S, Chetry M, Huang K, Peng Y, Wang J, Wang J, Zhou Y, Shen Y, Xue Y, Ji K (2018). Biosci Rep.

[129] Carmosino M, Procino G, Nicchia GP, Mannucci R, Verbavatz JM, Gobin R, Svelto M, Valenti G (2001). Histamine treatment induces rearrangements of orthogonal arrays of particles (OAPs) in human AQP4-expressing gastric cells. J Cell Biol.

[130] Fukuhara S, Matsuzaki J, Tsugawa H, Masaoka T, Miyoshi S, Mori H, Fukushima Y, Yasui M, Kanai T, Suzuki H (2014). Mucosal expression of aquaporin-4 in the stomach of histamine type 2 receptor knockout mice and Helicobacter pylori-infected mice. J Gastroenterol Hepatol.

[131] Matsuzaki J, Suzuki H, Minegishi Y, Sugai E, Tsugawa H, Yasui M, Hibi T (2010). Acid suppression by proton pump inhibitors enhances aquaporin-4 and KCNQ1 expression in gastric fundic parietal cells in mouse. Dig Dis Sci.

[132] Takeda T, Asaoka D, Tajima Y, Matsumoto K, Takeda N, Hiromoto T, Okubo S, Saito H, Aoyama T, Shibuya T, Sakamoto N, Hojo M, Osada T, Nagahara A, Yao T, Watanabe S (2017). Hemorrhagic polyps formed like fundic gland polyps during long-term proton pump inhibitor administration. Clin J Gastroenterol.

[133] Xu H, Zhang Y, Wei W, Shen L, Wu W (2009). Differential expression of aquaporin-4 in human gastric normal and cancer tissues. Gastroenterol Clin Biol.

[134] Thiagarajah JR, Zhao D, Verkman AS (2007). Impaired enterocyte proliferation in aquaporin-3 deficiency in mouse models of colitis. Gut.

[135] Zhu Y, Wang Y, Teng W, Shan Y, Yi S, Zhu S, Li Y (2019). Role of aquaporin-3 in intestinal injury induced by sepsis. Biol Pharm Bull.

[136] Hong Y, Chen Z, Li N, Zhang M (2020). Prognostic value of serum aquaporin-1, aquaporin-3 and galectin-3 for young patients with colon cancer. Ann Clin Biochem.

[137] Li A, Lu D, Zhang Y, Li J, Fang Y, Li F, Sun J (2013). Critical role of aquaporin-3 in epidermal growth factor-induced migration of colorectal carcinoma cells and its clinical significance. Oncol Rep.

[138] Kang BW, Kim JG, Lee SJ, Chae YS, Jeong JY, Yoon GS, Park SY, Kim HJ, Park JS, Choi GS, Jeong JY (2015). Expression of aquaporin-1, aquaporin-3, and aquaporin-5 correlates with nodal metastasis in colon cancer. Oncology.

[139] Hardin JA, Wallace LE, Wong JF, O'Loughlin EV, Urbanski SJ, Gall DG, MacNaughton WK, Beck PL (2004). Aquaporin expression is downregulated in a murine model of colitis and in patients with ulcerative colitis, Crohn’s disease and infectious colitis. Cell Tissue Res.

[140] Zhao G, Li J, Wang J, Shen X, Sun J (2014). Aquaporin 3 and 8 are down-regulated in TNBS-induced rat colitis. Biochem Biophys Res Commun.

[141] Su CG, Wen X, Bailey ST, Jiang W, Rangwala SM, Keilbaugh SA, Flanigan A, Murthy S, Lazar MA, Wu GD (1999). A novel therapy for colitis utilizing PPAR-gamma ligands to inhibit the epithelial inflammatory response. J Clin Invest.

[142] Lytle C, Tod TJ, Vo KT, Lee JW, Atkinson RD, Straus DS (2005). The peroxisome proliferator-activated receptor gamma ligand rosiglitazone delays the onset of inflammatory bowel disease in mice with interleukin 10 deficiency. Inflamm Bowel Dis.

[143] Zhang H, Du WB, Guo XM, Wang LK, Cheng JM, Wei LJ (2020). Identification of the AQP8-miR-92a network associated with the aggressive traits of colorectal cancer. Biochem Biophys Res Commun.

[144] O’Brien SJ, Kalbflesich T, Srivastava S, Pan J, Rai S, Petras RE, Ronquillo N, Polk HC, Galandiuk S (2021). Decreased tumoral expression of colon-specific water channel aquaporin 8 is associated with reduced overall survival in colon adenocarcinoma. Dis Colon Rectum.

[145] Fliegel L (2008). Molecular biology of the myocardial Na+/H+ exchanger. J Mol Cell Cardiol.

[146] Xu H, Ghishan FK, Kiela PR (2018). SLC9 gene family: function, expression, and regulation. Compr Physiol.

[147] Rossmann H, Sonnentag T, Heinzmann A, Seidler B, Bachmann O, Vieillard-Baron D, Gregor M, Seidler U (2001). Differential expression and regulation of Na(+)/H(+) exchanger isoforms in rabbit parietal and mucous cells. Am J Physiol Gastrointest Liver Physiol.

[148] Czepan M, Rakonczay Z, Varro A, Steele I, Dimaline R, Lertkowit N, Lonovics J, Schnur A, Biczo G, Geisz A, Lazar G, Simonka Z, Venglovecz V, Wittmann T, Hegyi P (2012). NHE1 activity contributes to migration and is necessary for proliferation of human gastric myofibroblasts. Pflugers Arch.

[149] Vor P, der Nolte A, Chodisetti G, Yuan Z, Busch F, Riederer B, Luo M, Yu Y, Menon MB, Schneider A, Stripecke R, Nikolovska K, Yeruva S, Seidler U (2017). Na(+) /H(+) exchanger NHE1 and NHE2 have opposite effects on migration velocity in rat gastric surface cells. J Cell Physiol.

[150] Miles LF, Coulson TG, Larsen T, Burbury KL, Story DA, Bellomo R (2020). Associations between preoperative inflammatory hyperferritinaemia and outcomes after major abdominal surgery. Br J Anaesth.

[151] Xue L, Aihara E, Wang TC, Montrose MH (2011). Trefoil factor 2 requires Na/H exchanger 2 activity to enhance mouse gastric epithelial repair. J Biol Chem.

[152] Engevik KA, Hanyu H, Matthis AL, Zhang T, Frey MR, Oshima Y, Aihara E, Montrose MH (2019). Trefoil factor 2 activation of CXCR4 requires calcium mobilization to drive epithelial repair in gastric organoids. J Physiol.

[153] Matthis AL, Kaji I, Engevik KA, Akiba Y, Kaunitz JD, Montrose MH, Aihara E (2020). Deficient active transport activity in healing mucosa after mild gastric epithelial damage. Dig Dis Sci.

[154] Moeser AJ, Nighot PK, Ryan KA, Wooten JG, Blikslager AT (2006). Prostaglandin-mediated inhibition of Na+/H+ exchanger isoform 2 stimulates recovery of barrier function in ischemia-injured intestine. Am J Physiol Gastrointest Liver Physiol.

[155] Sakakibara A, Furuse M, Saitou M, Ando-Akatsuka Y, Tsukita S (1997). Possible involvement of phosphorylation of occludin in tight junction formation. J Cell Biol.

[156] Xu H, Li J, Chen H, Wang C, Ghishan FK (2013). NHE8 plays important roles in gastric mucosal protection. Am J Physiol Gastrointest Liver Physiol.

[157] Liu C, Xu H, Zhang B, Johansson ME, Li J, Hansson GC, Ghishan FK (2013). NHE8 plays an important role in mucosal protection via its effect on bacterial adhesion. Am J Physiol Cell Physiol.

[158] Hansson GC, Johansson ME (2010). The inner of the two Muc2 mucin-dependent mucus layers in colon is devoid of bacteria. Gut Microbes.

[159] van Klinken BJ, Einerhand AW, Duits LA, Makkink MK, Tytgat KM, Renes IB, Verburg M, Buller HA, Dekker J (1999). Gastrointestinal expression and partial cDNA cloning of murine Muc2. Am J Physiol.

[160] Van der Sluis M, De Koning BA, De Bruijn AC, Velcich A, Meijerink JP, Van Goudoever JB, Buller HA, Dekker J, Van Seuningen I, Renes IB, Einerhand AW (2006). Muc2-deficient mice spontaneously develop colitis, indicating that MUC2 is critical for colonic protection. Gastroenterology.

[161] Sun Z, Luan S, Yao Y, Qin T, Xu X, Shen Z, Yao R, Yue L (2020). NHE1 mediates 5-Fu resistance in gastric cancer via STAT3 signaling pathway. Onco Targets Ther.

[162] Liu HF, Teng XC, Zheng JC, Chen G, Wang XW (2008). Effect of NHE1 antisense gene transfection on the biological behavior of SGC-7901 human gastric carcinoma cells. World J Gastroenterol.

[163] Aihara E, Matthis AL, Karns RA, Engevik KA, Jiang P, Wang J, Yacyshyn BR, Montrose MH (2016). Epithelial regeneration after gastric ulceration causes prolonged cell-type alterations. Cell Mol Gastroenterol Hepatol.

[164] Roginiel AC, Kohut DL, Kaur S, Saleh AM, Weber T, Geibel P, Singh H, Geibel JP (2013). Effect of NSAIDs on Na(+)/H(+) exchanger activity in rat colonic crypts. Am J Physiol Cell Physiol.

[165] Soleiman AA, Thameem F, Khan I (2017). Mechanism of down regulation of Na-H exchanger-2 in experimental colitis. PLoS ONE.

[166] Rocha F, Musch MW, Lishanskiy L, Bookstein C, Sugi K, Xie Y, Chang EB (2001). IFN-gamma downregulates expression of Na(+)/H(+) exchangers NHE2 and NHE3 in rat intestine and human Caco-2/bbe cells. Am J Physiol Cell Physiol.

[167] Amin MR, Orenuga T, Tyagi S, Dudeja PK, Ramaswamy K, Malakooti J (2011). Tumor necrosis factor-alpha represses the expression of NHE2 through NF-kappaB activation in intestinal epithelial cell model, C2BBe1. Inflamm Bowel Dis.

[168] Rajendran VM, Nanda Kumar NS, Tse CM, Binder HJ (2015). Na-H exchanger isoform-2 (NHE2) mediates butyrate-dependent Na+ absorption in dextran sulfate sodium (DSS)-induced colitis. J Biol Chem.

[169] Bernardazzi C, Xu H, Tong H, Laubitz D, Figliuolo da Paz V, Curiel L, Ghishan FK (2020). An indisputable role of NHE8 in mucosal protection. Am J Physiol Gastrointest Liver Physiol.

[170] Xu H, Li J, Chen H, Ghishan FK (2019). NHE8 deficiency promotes colitis-associated cancer in mice via expansion of Lgr5-expressing cells. Cell Mol Gastroenterol Hepatol.

[171] Lei X, Cai L, Li X, Xu H, Geng C, Wang C (2018). Up-regulation of NHE8 by somatostatin ameliorates the diarrhea symptom in infectious colitis mice model. Korean J Physiol Pharmacol.

[172] Li X, Cai L, Xu H, Geng C, Lu J, Tao L, Sun D, Ghishan FK, Wang C (2016). Somatostatin regulates NHE8 protein expression via the ERK1/2 MAPK pathway in DSS-induced colitis mice. Am J Physiol Gastrointest Liver Physiol.

[173] Wang C, Xu H, Chen H, Li J, Zhang B, Tang C, Ghishan FK (2011). Somatostatin stimulates intestinal NHE8 expression via p38 MAPK pathway. Am J Physiol Cell Physiol.

[174] Han J, Lee SH, Giebisch G, Wang T (2016). Potassium channelopathies and gastrointestinal ulceration. Gut Liver.

[175] Aziz Q, Li Y, Anderson N, Ojake L, Tsisanova E, Tinker A (2017). Molecular and functional characterization of the endothelial ATP-sensitive potassium channel. J Biol Chem.

[176] Kwon SC, Kim JH (2021). Gastroprotective effects of irsogladine maleate on ethanol/hydrochloric acid induced gastric ulcers in mice. Korean J Intern Med.

[177] Magierowska K, Korbut E, Hubalewska-Mazgaj M, Surmiak M, Chmura A, Bakalarz D, Buszewicz G, Wojcik D, Sliwowski Z, Ginter G, Gromowski T, Kwiecien S, Brzozowski T, Magierowski M (2019). Oxidative gastric mucosal damage induced by ischemia/reperfusion and the mechanisms of its prevention by carbon monoxide-releasing tricarbonyldichlororuthenium (II) dimer. Free Radic Biol Med.

[178] Kaufhold MA, Krabbenhoft A, Song P, Engelhardt R, Riederer B, Fahrmann M, Klocker N, Beil W, Manns M, Hagen SJ, Seidler U (2008). Localization, trafficking, and significance for acid secretion of parietal cell Kir4.1 and KCNQ1 K+ channels. Gastroenterology.

[179] Nguyen N, Kozer-Gorevich N, Gliddon BL, Smolka AJ, Clayton AH, Gleeson PA, van Driel IR (2013). Independent trafficking of the KCNQ1 K+ channel and H+-K+-ATPase in gastric parietal cells from mice. Am J Physiol Gastrointest Liver Physiol.

[180] Jain RN, Brunkan CS, Chew CS, Samuelson LC (2006). Gene expression profiling of gastrin target genes in parietal cells. Physiol Genomics.

[181] Grahammer F, Herling AW, Lang HJ, Schmitt-Graff A, Wittekindt OH, Nitschke R, Bleich M, Barhanin J, Warth R (2001). The cardiac K+ channel KCNQ1 is essential for gastric acid secretion. Gastroenterology.

[182] Schubert ML (2011). Gastric secretion. Curr Opin Gastroenterol.

[183] Dong H, Smith A, Hovaida M, Chow JY (2006). Role of Ca2+-activated K+ channels in duodenal mucosal ion transport and bicarbonate secretion. Am J Physiol Gastrointest Liver Physiol.

[184] Sheikh IA, Koley H, Chakrabarti MK, Hoque KM (2013). The Epac1 signaling pathway regulates Cl- secretion via modulation of apical KCNN4c channels in diarrhea. J Biol Chem.

[185] Bowley KA, Morton MJ, Hunter M, Sandle GI (2003). Non-genomic regulation of intermediate conductance potassium channels by aldosterone in human colonic crypt cells. Gut.

[186] Ayabe T, Wulff H, Darmoul D, Cahalan MD, Chandy KG, Ouellette AJ (2002). Modulation of mouse Paneth cell alpha-defensin secretion by mIKCa1, a Ca2+-activated, intermediate conductance potassium channel. J Biol Chem.

[187] Chandy KG, Wulff H, Beeton C, Pennington M, Gutman GA, Cahalan MD (2004). K+ channels as targets for specific immunomodulation. Trends Pharmacol Sci.

[188] Ghanshani S, Wulff H, Miller MJ, Rohm H, Neben A, Gutman GA, Cahalan MD, Chandy KG (2000). Up-regulation of the IKCa1 potassium channel during T-cell activation. Molecular mechanism and functional consequences. J Biol Chem.

[189] Koch Hansen L, Sevelsted-Moller L, Rabjerg M, Larsen D, Hansen TP, Klinge L, Wulff H, Knudsen T, Kjeldsen J, Kohler R (2014). Expression of T-cell KV1.3 potassium channel correlates with pro-inflammatory cytokines and disease activity in ulcerative colitis. J Crohns Colitis.

[190] Toroudi HP, Rahgozar M, Bakhtiarian A, Djahanguiri B (1999). Potassium channel modulators and indomethacin-induced gastric ulceration in rats. Scand J Gastroenterol.

[191] Rahgozar M, Pazokitoroudi H, Bakhtiarian A, Djahanguiri B (2001). Diazoxide, a K(ATP) opener, accelerates restitution of ethanol or indomethacin-induced gastric ulceration in rats independent of polyamines. J Gastroenterol Hepatol.

[192] Menozzi A, Pozzoli C, Poli E, Passeri B, Gianelli P, Bertini S (2011). Diazoxide attenuates indomethacin-induced small intestinal damage in the rat. Eur J Pharmacol.

[193] Sun HZ, Zheng S, Lu K, Hou FT, Bi JX, Liu XL, Wang SS (2017). Hydrogen sulfide attenuates gastric mucosal injury induced by restraint water-immersion stress via activation of KATP channel and NF-kappaB dependent pathway. World J Gastroenterol.

[194] Tomisato W, Tanaka K, Katsu T, Kakuta H, Sasaki K, Tsutsumi S, Hoshino T, Aburaya M, Li D, Tsuchiya T, Suzuki K, Yokomizo K, Mizushima T (2004). Membrane permeabilization by non-steroidal anti-inflammatory drugs. Biochem Biophys Res Commun.

[195] Ashby WB, Humphreys J, Smith SJ (1965). Small-bowel ulceration induced by potassium chloride. Br Med J.

[196] Boley SJ, Schultz L, Krieger H, Schwartz S, Elguezabal A, Allen AC (1965). Experimental evaluation of thiazides and potassium as a cause of small-bowel ulcer. JAMA.

[197] Leijonmarck CE, Raf L (1985). Ulceration of the small intestine due to slow-release potassium chloride tablets. Acta Chir Scand.

[198] Elso CM, Lu X, Culiat CT, Rutledge JC, Cacheiro NL, Generoso WM, Stubbs LJ (2004). Heightened susceptibility to chronic gastritis, hyperplasia and metaplasia in Kcnq1 mutant mice. Hum Mol Genet.

[199] Yang Z, Yuan L, Yang L, Peng S, Yang P, He X, Bao G (2021). Association study between KCNQ1 and KCNQ1OT1 genetic polymorphisms and gastric cancer susceptibility and survival in a Chinese Han population: a case-control study. Ann Transl Med.

[200] Than BL, Goos JA, Sarver AL, O'Sullivan MG, Rod A, Starr TK, Fijneman RJ, Meijer GA, Zhao L, Zhang Y, Largaespada DA, Scott PM, Cormier RT (2014). The role of KCNQ1 in mouse and human gastrointestinal cancers. Oncogene.

[201] Zhang J, Zhao Z, Zu C, Hu H, Shen H, Zhang M, Wang J (2013). Atrial natriuretic peptide modulates the proliferation of human gastric cancer cells via KCNQ1 expression. Oncol Lett.

[202] Al-Hazza A, Linley JE, Aziz Q, Maclennan KA, Hunter M, Sandle GI (2012). Potential role of reduced basolateral potassium (IKCa3.1) channel expression in the pathogenesis of diarrhoea in ulcerative colitis. J Pathol.

[203] Di L, Srivastava S, Zhdanova O, Ding Y, Li Z, Wulff H, Lafaille M, Skolnik EY (2010). Inhibition of the K+ channel KCa3.1 ameliorates T cell-mediated colitis. Proc Natl Acad Sci USA.

[204] Suss C, Broncy L, Pollinger K, Kunst C, Gulow K, Muller M, Wolfel G (2020). KCNN4 expression is elevated in inflammatory bowel disease: this might be a novel marker and therapeutic option targeting potassium channels. J Gastrointestin Liver Dis.

[205] Simms LA, Doecke JD, Roberts RL, Fowler EV, Zhao ZZ, McGuckin MA, Huang N, Hayward NK, Webb PM, Whiteman DC, Cavanaugh JA, McCallum R, Florin TH, Barclay ML, Gearry RB, Merriman TR, Montgomery GW, Radford-Smith GL (2010). KCNN4 gene variant is associated with ileal Crohn’s disease in the Australian and New Zealand population. Am J Gastroenterol.

[206] Ibrahim S, Dakik H, Vandier C, Chautard R, Paintaud G, Mazurier F, Lecomte T, Gueguinou M, Raoul W (2019). Expression profiling of calcium channels and calcium-activated potassium channels in colorectal cancer. Cancers (Basel).

[207] Lai W, Liu L, Zeng Y, Wu H, Xu H, Chen S, Chu Z (2013). KCNN4 channels participate in the EMT induced by PRL-3 in colorectal cancer. Med Oncol.

[208] Pillozzi S, D'Amico M, Bartoli G, Gasparoli L, Petroni G, Crociani O, Marzo T, Guerriero A, Messori L, Severi M, Udisti R, Wulff H, Chandy KG, Becchetti A, Arcangeli A (2018). The combined activation of KCa3.1 and inhibition of Kv11.1/hERG1 currents contribute to overcome Cisplatin resistance in colorectal cancer cells. Br J Cancer.

